# Ropiness in Bread—A Re-Emerging Spoilage Phenomenon

**DOI:** 10.3390/foods11193021

**Published:** 2022-09-29

**Authors:** Nicola Pacher, Johanna Burtscher, Sophia Johler, Danai Etter, Denisse Bender, Lars Fieseler, Konrad J. Domig

**Affiliations:** 1 Institute of Food Science, Department of Food Science and Technology, University of Natural Resources and Life Sciences, Vienna, Muthgasse 18, 1190 Vienna, Austria; 2 Institute for Food Safety and Hygiene, Vetsuisse Faculty, University of Zurich, Winterthurerstr. 272, 8057 Zurich, Switzerland; 3 Institute of Food and Beverage Innovation, ZHAW Zurich University of Applied Sciences, Einsiedlerstrasse 31, 8820 Wädenswil, Switzerland

**Keywords:** *Bacillus* spp., bread, rope spoilage, wheat

## Abstract

As bread is a very important staple food, its spoilage threatens global food security. Ropy bread spoilage manifests in sticky and stringy degradation of the crumb, slime formation, discoloration, and an odor reminiscent of rotting fruit. Increasing consumer demand for preservative-free products and global warming may increase the occurrence of ropy spoilage. *Bacillus amyloliquefaciens*, *B. subtilis*, *B. licheniformis*, the *B. cereus* group, *B. pumilus*, *B. sonorensis*, *Cytobacillus firmus*, *Niallia circulans*, *Paenibacillus polymyxa*, and *Priestia megaterium* were reported to cause ropiness in bread. Process hygiene does not prevent ropy spoilage, as contamination of flour with these *Bacillus* species is unavoidable due to their occurrence as a part of the endophytic commensal microbiota of wheat and the formation of heat-stable endospores that are not inactivated during processing, baking, or storage. To date, the underlying mechanisms behind ropy bread spoilage remain unclear, high-throughput screening tools to identify rope-forming bacteria are missing, and only a limited number of strategies to reduce rope spoilage were described. This review provides a current overview on (i) routes of entry of *Bacillus* endospores into bread, (ii) bacterial species implicated in rope spoilage, (iii) factors influencing rope development, and (iv) methods used to assess bacterial rope-forming potential. Finally, we pinpoint key gaps in knowledge and related challenges, as well as future research questions.

## 1. Introduction

Bread is a very important staple food. Cereals are, therefore, considered some of the most important agricultural products and occupy approximately 60% of all cultivated land. More than 50% of the world’s daily caloric intake is directly derived from cereal grains and their products, such as bread [1,2]. In its simplest form, bread comprises a mixture of water and flour to produce a batter or dough, which may be fermented prior to baking, frying, or steaming, resulting in a remarkably versatile product [3,4]. Bread preparation involves various modification processes of the cereal’s seeds to reduce noxious or indigestible compounds and facilitate the digestion of valuable components present. Indigestible and abrasive glumes of hulled cereals are removed by dehusking, and milling breaks up the grain’s starch-containing endosperm tissue [4]. High baking temperatures facilitate protein digestion, e.g., by denaturation of protease inhibitors. Taste is enhanced by the Maillard reaction, in addition to better starch accessibility by gelatinization [3,4,5].

Spoilage of bread and other bakery products can manifest as inanimate physical and chemical spoilage with moisture loss or rancidity, or in the form of animate spoilage due to growth of molds or bacteria [6]. The spoilage potential of bakery products is dependent on their acidity (high: pH < 4.6; low: pH 4.6–7; non-acidic: pH > 7) and water activity (high: a_w_ > 0.85; intermediate: a_w_ = 0.6 to 0.85; low: a_w_ < 0.6), with high moisture and low acidity products being the most susceptible to microbiological spoilage [6]. By the end of the 19th century, spore-forming bacilli were identified as causative agents of ropy bread spoilage [7]. Besides molds, *Bacillus* spp., originating from raw materials or bakery equipment, are among the most important spoilage agents of non-acidified white and wholemeal bread [8,9]. At the beginning of the 20th century, research efforts focused on the control of rope-spoilage organisms and the inhibition of their germination and outgrowth through preservatives, such as lactic acid, acetic acid, or propionic acid [10,11,12]. The bakery industry is now heavily dependent on preservatives to control ropey spoilage. Generally, sodium, potassium or calcium salts of propionic and sorbic acid are used as chemical preservatives in bakery products [13]. Recently, however, increased demand for preservative-free “clean label” products and the inclusion of whole-grain flours [13,14], in combination with global warming and rising ambient temperatures, may lead to more frequent occurrence of bread spoilage by rope-forming bacilli. 

**Figure 1:**
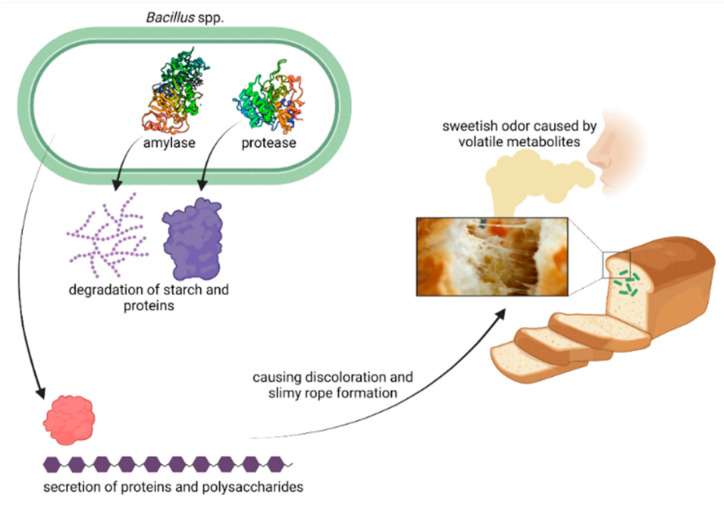
Mechanisms of rope formation, discoloration, odor development, and changes in bread structure. Created with BioRender.com.

*Bacillus* spp. is a gram-positive, rod-shaped, aerobic or facultative anaerobic, motile, and endospore-forming bacterium [15]. The presence of endospores in raw ingredients and foods is practically impossible to prevent due to the ubiquitous occurrence of *Bacillus* spp. in nature [16]. *Bacillus* endospores are not only heat stable and able to survive baking in the center of the bread crumb, but they are also highly resistant to desiccation, radiation, and chemical agents [12,17]. Intracellular endospore formation is triggered by nutrient starvation, with subsequent cell lysis and the release of spores [18]. If conditions are favorable, for instance temperatures above 25 °C in combination with an a_w_ ≥ 0.95 and pH > 5, spore germination and growth can lead to spoilage (Table in Section 3) [19]. Rope formation may occur in localized high-moisture areas inside the bread loaf [8]. Ropiness is associated with a patchy discoloration and a stringy bread crumb, and characterized by an unpleasant sweetish odor resembling rotting melons or pineapples that is caused by the release of volatile compounds including diacetyl, acetoin, acetaldehyde, and isovaleraldehyde (Figure 1) [8,20,21]. In advanced stages, the bread crumb can be almost liquefied and forms long, silky, web-like strands when pulled apart, as depicted in Figure 1, giving rise to the designation of rope spoilage [8,22,23]. This phenomenon mostly occurs in high-moisture bakery products during summer months or countries with moist and hot climates [6,24]. The production of extracellular slimy polysaccharides and proteins, as well as the production of proteolytic and amylolytic enzymes that degrade the bread crumb, is characteristic of rope-forming bacterial species (Figure 1) [9,24,25,26].

The aim of this review is to provide an overview of the current state of knowledge on rope spoilage in bread and pinpoint research gaps hindering a better understanding of this spoilage phenomenon. To this end, (i) routes of *Bacillus* endospores into bread, (ii) the diversity of bacterial species involved in rope spoilage, (iii) the variety of factors influencing rope development, as well as (iv) available methods used to assess bacterial rope-forming potential and their limitations are described.

## 2. The Route of Endospores into the Bakery Environment

**Figure 2:**
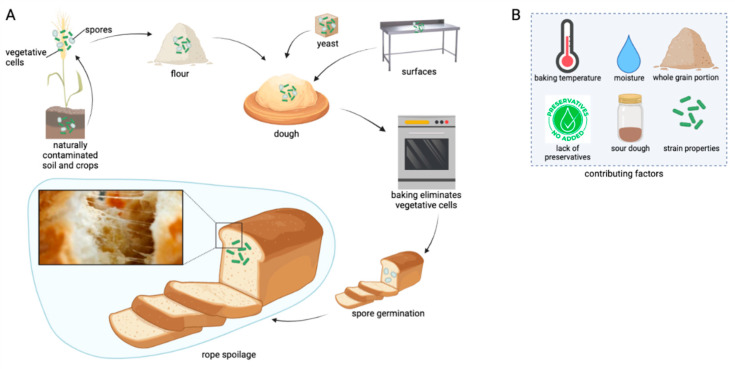
The route of endospores into bread (**A**) and influencing factors for the development of rope spoilage (**B**). Created with BioRender.com.

*Bacillus* spp. are ubiquitous in nature and form symbiotic communities with different types of plants [27]. Soil is considered as the primary endospore reservoir with concentrations of up to 10^6^ spores/g, thus representing a major route of entry for *Bacillus* spp. into the food chain (Figure 2A) [28]. Contamination levels of cereal grains and successive products may vary because of the influence of the cereal microbiota, which is dependent on growth conditions, including the location of crop growth and atmospheric conditions, such as precipitation levels and relative humidity [2,29,30,31]. Studies suggest that wheat originating from hotter, wetter areas generally carries higher microbial loads, as wheat grown under dry and warm wheatear conditions exhibited bacterial counts of 5.7 log CFU/g in contrast to 8.1 log CFU/g for wheat grown under unusually wet conditions [31,32]. Further, Sabillón et al. [33] observed lower microbial loads on wheat in areas where relative humidity levels were below 55%, and the temperatures were lower than 13.7 °C and higher than 31.5 °C.

Cereal quality is affected by lodging (i.e., displacement of crop stems from their upright position) due to adverse environmental conditions and improper crop management practices, such as excessive nitrogen application or high planting density. Lodged crops may come into direct contact with moist soil, thus leading to rapidly increasing microbial loads and causing grain-quality deterioration along the processing line [29,34,35].

Generally, dry grains are stored under conditions that do not allow bacterial proliferation. Therefore, bacterial spores do not germinate until the grains moisture content and temperature increase [36]. If the overall moisture content increases to an a_w_ of 0.68–0.75, molds are able to grow. Their metabolic activities can raise the grain temperature to 30–40 °C. Moisture evaporates from these warm areas and condenses in cooler areas of the bulk-stored grain [37]. Consequently, the excessive moisture and temperature increase within the grain may lead to bacterial spore germination during cereal storage.

During the milling process, foreign materials such as soil, plant-based remains, insects, and animal excrement are removed, and cereal kernels are broken, reduced, ground, and sifted [31,38]. During this process, the endosperm is separated from the outer layers of the grain, ground to semolina, and further into flour [32,39]. One of the most critical steps is the tempering of grains before milling. Higher mesophilic counts were found more frequently in tempered wheat kernels [38]. Berghofer et al. [32] deduced that this was due to cross-contamination from poorly sanitized tempering bins rather than microbial growth, as high microbial counts were found in tempering bins. Sabillón et al. [38] reported aerobic plate counts (APCs) of 4.8 ± 1.4 CFU/cm^2^ in the cleaning and tempering equipment of a milling plant. Microbial populations are typically confined to the outer layers of cereal grains, such as the husk, between the husk and bran, or within the bran’s pericarp tissue [2,40]. The microbial load of milling products is lowest when husk particles are reduced to a minimum; thus, flours containing the inner semolina fraction exhibit lower microbial loads compared with bran, wheat germ, and pollard [29,32]. Berghofer et al. [32] reported *Bacillus* spp. counts of 10^4^ CFU/g in Australian wheat. As wholemeal flour contains a larger amount of grain outer layers or fiber residues, its microbial load is considerably higher compared with those of more refined flours [41]. Nevertheless, even the best milling procedures do not ensure complete separation of the starchy endosperm from other kernel constituents [42]. In addition, microbial contaminants present in the raw material are often redistributed among milling products, possibly resulting in a refined flour of inferior microbial quality [30]. As none of the milling operations include a microbial-reduction step, levels of microorganisms in raw cereals combined with poor milling hygiene directly affect the microbial quality of flour [12,29,38].

Due to its low a_w_ (<0.60), flour does not support bacterial growth and is regarded as a microbiologically safe product [32]. Nevertheless, bacterial spore concentrations in flours can exceed 10^3^ spores/g and spores remain dormant for long periods of time [28]. Consequently, high loads of bacterial spores prominently endanger the quality and shelf life of bread [28,43].

Baker’s yeasts are also known to be sources of contamination with spoilage bacteria, as studies showed that it is almost impossible to obtain bacteria-free commercial yeast [22,44]. Due to the large scale of industrial yeast fermentation, spoilage bacteria are able to enter the fermentation cycle at several production steps, as maintaining sterile conditions throughout the whole process is unachievable [45]. Consequently, baker’s yeast is considered an important introduction vector of vegetative bacteria and spores into the baking environment (Figure 2A) [46]. Viljoen and coworkers suggested that compressed yeast might represent the main source of bacterial contamination in bread doughs, as total aerobic counts of up to 10^8^ CFU/g in baker’s yeast were observed [47,48]. Several studies reported the presence of different rope-associated spore formers in raw materials, with baker’s yeast showing high *Bacillus* spp. spore counts of 5.15 log spores/g [49]. Reale et al. [45] found vegetative *Bacillus* spp. in concentrations of 3.9 ± 0.1 log CFU/g in compressed baker’s yeast, whereas the spore concentrations were distributed around 2 log spores/g in compressed and dried yeast samples, respectively.

Besides their occurrence in raw materials, rope-associated spores may also be present on equipment surfaces (Figure 2A), as well as in the bakery atmosphere [26,50]. Rope spoilage can result from inadequately cleaned and sanitized equipment, as spores may contaminate mixers, dough bowls, pipelines, filters, water tanks, conveyor belts, slicing blades, and other equipment [12]. Bailey and Holy [49] showed that *Bacillus* spore contamination of pre-baking food contact surfaces was not higher than 2.5 log CFU/swab, concluding that equipment surface contamination did not contribute significantly to the overall spore contamination of the bakery environment. In 1997, Viljoen and Holy investigated the microbial ecology of a commercial bread production line. According to their data, the APCs of equipment surfaces ranged between 7 and 5 log CFU/swab with high species variability. From 316 bacterial isolates, 50% were identified as *Bacillus* spp. [48]. While most of the studies focus on the occurrence of rope-forming bacteria in the bakery environment, few studies quantified bacilli in bread. Rosenkvist [24] sampled white and wholemeal wheat loaves baked without preservatives from different retail bakers. *Bacillus* spp. counts above 10^6^ CFU/g after two days of storage at ambient summer temperatures of 25–30 °C were consistently enumerated.

## 3. The Diversity of Species Inducing Rope Spoilage in Bread

Watkins [11] and Cohn et al. [10] identified *Bacillus* spp. as the main cause of ropy spoilage of bakery products, but recent literature on the matter is scarce. Considering recent changes in taxonomy and the microbiological and molecular biological methodologies used, attribution of strains to some of the species might not be consistent with current taxonomic frameworks and must be interpreted with caution. Table 1 provides an overview over the different bacterial species associated with rope spoilage. Amos and Kent-Jones [7] attributed rope formation exclusively to strains belonging to the *B. mesentericus* group, later known as *Bacillus subtilis* ssp. *subtilis* [15]. Collins et al. [50] characterized 102 *Bacillus* isolates from ropy bread, bakery equipment, and raw materials using standard biochemical tests and morphological characteristics. Of the 102 *Bacillus* isolates, 63.7% were identified as *B. subtilis*, 24.5% as *B. licheniformis*, 8.8% as *B. pumilus*, two isolates as members of the *B. cereus* group, and one as *B. firmus*, today known as *Cytobacillus firmus* [50,51]. Subsequent characterization of the same isolates using an API 50CH identification kit led to identification of 40.8% of the isolates as *B. subtilis*, 17.4% as *B. amyloliquefaciens*, 17.4% *B. licheniformis*, 20.4% *B. pumilus*, two isolates as members of the *B. cereus* group, and one as *B. circulans*, which today is known as *N. circulans* [50,52]. In a subsequent study, phenotypic profiles of these 102 strains were created using API 50CH and API 20E test strips and biochemical tests [53]. Dykes et al. [53] also analyzed their results using the single-matching coefficient with single-linkage clustering to construct simplified dendrograms. *B. subtilis* (57.7%) represented the greatest proportion of *Bacillus* strains isolated from ropy bread. However, the reference strains *B. subtilis* ATCC 6633, now known as *B. spizizenii*; *B. licheniformis* ATCC 14580; *B. megaterium* ATCC 12872, today known as *P. megaterium*; *B. macerans* ATCC 8244, today known as *P. macerans*; and *B. sphaericus* ATCC 14577, today known as *Lysinibacillus sphaericus*, did not cluster with bakery-associated isolates [52,53,54,55]. Three highly heat-resistant reference strains clustering with ropy bread isolates were those originating from heat control studies, namely *B. cereus* ATCC 7004, *B. subtilis* NCTC 10073, and *B. subtilis* ATCC 9372. Today, the last two strains are known as *B. atrophaeus* [56]. Dykes et al. [53] concluded that high baking temperatures exerted significant selection pressure on spore heat resistance and consequently on isolate diversity. Further, the ropy bread isolates also demonstrated high phenotypic similarity, with similar biochemical and morphological characteristics. Rosenkvist [24] identified *B. subtilis*, *B. licheniformis*, *B. pumilus*, and the *B. cereus* group as the major contaminants of white and wholemeal wheat loaves and its raw materials, but only *B. subtilis* could be associated with ropiness [24].

Pepe et al. [25] isolated 61 cultures of gram-positive spore formers from 32 ropy samples of industrial and artisanal white wheat high-type breads (height ≥ 10 cm), and 24 ropy samples of artisanal white wheat low-type breads (height ≤ 10 cm). The isolates were identified as *B. subtilis* using the Norris et al. [67] dichotomous key. Of those presumptive *B. subtilis* strains, 34 were further characterized by RAPD-PCR, showing 11 different patterns. The 34 strains were also grouped into four profiles according to sequence-specific separation of the V3 region of 16S rDNA by denaturing gradient gel electrophoresis (DGGE) analysis. Molecular identification, by sequencing of the V3 region of 16S rDNA, was then performed for 10 strains representing all DGGE and RAPD-PCR patterns. Sequences from the four DGGE patterns were closely related to *B. subtilis* and *B. licheniformis*; the *B. cereus* group; *B. clausii*, today known as *Alkalihalobacillus clausii*; and *B. firmus*, today known as *C. firmus*, respectively [25,68]. 16S rDNA sequencing and RAPD-PCR were also used by Sorokulova et al. [63] in an attempt to assess the genetic diversity and involvement in bread spoilage of *Bacillus* strains. A total of 30 strains were isolated from flour and ropy bread. The bacteria were identified by phenotypic characteristics and 16S rDNA sequencing, with 24 *B. subtilis* isolates representing the predominant species, followed by 6 *B. licheniformis* isolates. From the isolated bacteria, 10 out of 15 *B. subtilis*, and 4 out of 6 *B. licheniformis* isolates, were able to cause rope spoilage in laboratory-baked bread. The RAPD analysis associated two *Bacillus* strains isolated from flour with rope spoilage in laboratory-baked bread [63].

Given the low accuracy of identification methods, such as API 50CH or API 20E test strips and biochemical tests, Valerio et al. [26] examined the diversity of spore-forming bacteria isolated from raw materials and bread by sequencing the 16S rDNA and the *gyrA* or *gyrB* genes, and using FT-NIR spectroscopy. Raw materials included semolina, grain, yeast, and baking improvers, which were characterized by high bacterial diversity, with microbial loads being as high as 100 spores/g. The working group isolated and characterized a total of 176 isolates. *B. amyloliquefaciens* was the most frequently isolated species (54%), followed by the *B. cereus* group (18%), *Paenibacillus* spp. (9%), *B. licheniformis* (7%), *B. subtilis* (3%), *B. pumilus* (2%), and *Pr. megaterium* (2%) [19,52]. Interestingly, 79% of these *B. amyloliquefaciens* isolates maintained their amylase activity and were able to cause rope spoilage, even after heat treatment at 100 °C. The same applied for 83% of B. subtilis, 67% of *B. pumilus* and *Pr. megaterium*, 38% of the *B. cereus* group, and 11% of *Paenibacillus* spp. isolates [19]. A similar prevalence study was also performed by Pereira et al. [41]. From 100 analyzed flour samples, a total of 327 different isolates were gained. Wholemeal flour showed the highest microbial load of amylase-producing strains and white bread flour the highest diversity. The amylase-producing strains were then identified via 16S rDNA sequencing as *B. licheniformis* (62%); *B. sonorensis* (20%); *B. cereus* group (11%); *B. subtilis* (2%); *B. pumilus* (2%); and *B. polymyxa* (2%), now known as *P. polymyxa* [41,60]. This prevalence study by Pereira et al. [41] is consistent with the findings of Ma et al. [65] in terms of *Bacillus* being detected as the predominant genus, with a relative abundance of 41%, in steamed wheat bread crumb. However, on the species level, results vary strongly. Ma et al. [66] determined *B. subtilis* as the dominant *Bacillus* species, while Valerio et al. [26] determined *B. amyloliquefaciens*, and Pereira et al. [41] *B. licheniformis*, as the predominant species.

A major problem when identifying species associated with rope spoilage is their close relationship and the choice of analytical methods. 16S rDNA sequencing alone, as used by Pereira et al. [41] and Ma et al. [66], is not able to differentiate closely related *Bacillus* species. Valerio et al. [26] combined 16S rDNA sequencing and sequencing of *gyrA*, which allowed further differentiation among *B. subtilis*, *B. amyloliquefaciens*, and *B. mojavensis*. The *gyrB* gene was used to discriminate between *B. pumilus* and *B. safensis*, and the ability to grow in the presence of 7% NaCl to then differentiate *B. licheniformis* (Table 1) from *B. safensis* [26]. Sadeghi et al. [69] tried to avoid sequencing of 16S rDNA entirely and developed a multiplex PCR assay to target the *IpaL* gene of *B. licheniformis* ATCC 9789 and the *aprE* gene of *B. subtilis* ATCC 6633, now known as *B. spizizenii*, in bread dough [51,69]. This assay was further developed into a qPCR with SYBR Green in order to detect *B. subtilis* in dough, although this is now obsolete, as it is no longer specific to *B. subtilis* [70].

The correct assessment of species diversity causing rope spoilage is further complicated by the everchanging taxonomy within the *Bacillus* genus and the taxonomic collapse of the *B. cereus* group [52,71]. Recently, Gupta et al. [52] proposed amending the genus *Bacillus* by restricting it to only members of the Subtilis and Cereus clades. In this context, many of the identified rope formers are representatives of the Subtilis clade, including *B. subtilis*, *B. amyloliquefaciens*, *B. pumilus*, and *B. sonorensis* [52]. Furthermore, *B. subtilis* once encompassed four subspecies, namely *B. subtilis* subsp. *subtilis*, *B. subtilis* subsp. *inaquosorum*, *B. subtilis* subsp. *spizizenii*, and *B. subtilis* subsp. *stercoris*. All four subspecies have an average genome size of 4.1 Mbp with an average G + C mole content of 43.8%. After genomic comparisons, these subspecies were promoted to species status, augmenting the Subtilis clade by three species with the type strains *B. inaquosorum* DSM 22,148, *B. spizizenii* DSM 15,029, and *B. stercoris* KCTC 33,554 [54]. Thus, the diversity of species potentially responsible for rope spoilage was increased further, and *B. subtilis* isolates involved in rope spoilage could potentially belong to these new species. However, there is no direct evidence of their involvement in rope spoilage.

## 4. Rope-Inducing Potential (RIP)

### 4.1. Methods of RIP Assessment

A standardized culture medium allowing analysis of the RIP of flours, based on the development of typical odor and pigment formation, was introduced by Russ et al. [72]. The authors suggested it correlated well with baking tests and proposed it as a suitable method for the detection of potential rope-developing flour [72]. Later studies demonstrated poor correlation between media inoculation and baking tests, therefore calling into question its suitability [73].

For the RIP assessment of different *Bacillus* isolates, Thompson et al. [23] proposed a direct inoculation method for bread slices. A slice of a loaf was placed into a petri dish, inoculated with 1 mL of overnight broth culture, and incubated at 30 °C for 96 h. After incubation, the bread slices were visually examined. The degree of rope spoilage was graded on a seven-level scale as no rope (−), questionable rope (±), slight rope (+), moderate rope (++), significant rope (+++), advanced rope (++++), and very advanced rope (+++++) [23]. The authors noted that the amount of rope produced by an isolate was not constant in all loaf types, and even fluctuated between batches of the same variety, thus hypothesizing that direct inoculation of loaves with bacterial culture did not allow assessment of the RIP of *Bacillus* isolates [23]. However, all bread types used in this study contained preservatives such as calcium propionate or vinegar, which impair bacterial growth.

This assay was frequently applied with minor adaptations, as it offers some insight into an isolate’s spoilage potential [25,26,41]. Briefly, the *Bacillus* isolates of interest are inoculated in 20 mL bread extract broth (BEB) and incubated overnight at 30 °C. BEB is prepared by homogenizing 100 g of white bread slices with 350 mL distilled water, subsequent filtration through a filter paper, and pH adjustment to 6.8 with 1 M NaOH prior to sterilization in an autoclave at 121 °C for 15 min. The inoculated broth is then divided into 5 mL aliquots. Depending on the assay, the aliquots are further heat treated at different temperatures (96/100 °C, 10 min) or are left untreated [25,41]. Acquired or laboratory-baked white bread slices, with or without preservatives, are transferred to petri dishes and sterilized in an autoclave (121 °C, 15 min) to inactivate any spore-forming bacteria already present in the bread [41,74]. Pereira et al. [41] performed preliminary experiments to ensure that moisture content, a_w_, and pH did not change during this treatment. After autoclaving, the inocula are distributed on the surface of the bread slices. The final concentration of spores per bread slice is adapted according to the assay. For instance, Pereira et al. [41] adjusted the spore concentrations to approximately 10^3^ spores/g. The inoculated slices are incubated at 30 °C, and rope development is evaluated daily and graded according to the scale developed by Thompson et al. [23]. The control slices are inoculated with an aliquot of 5 mL sterilized water [25,41].

### 4.2. Factors Influencing RIP

Volavsek et al. [73] argue that not only the numbers, but also the type of *Bacillus* spores, influence the RIP as some are more efficient in causing rope spoilage. Test baking doughs artificially inoculated with defined spore suspensions played a central role in estimating their heat resistance and sporulation, along with their growth and RIP. Rosenkvist [24] showed that even low spore levels in raw materials led to high cell counts. The authors enumerated *Bacillus* counts of 10^7^ CFU/g within the bread crumb after two days of storage [24].

The type of bread and internal temperature reached during baking, coupled with the endospore heat resistance, greatly influence endospore survival during the baking process (Figure 2B) [19]. For instance, Pepe et al. [25] noticed that high-type breads (height ≥ 10 cm) were more frequently affected by ropiness, suggesting that endospores survive the baking procedure in the middle of the bread crumb. Underbaking further promotes rope development, as a moist crumb greatly supports the outgrowth of bacterial spores [22]. This is one reason for the incidence of rope spoilage being significantly higher, compared with western countries, in South Africa, where consumer preferences are higher for warm, slightly underbaked brown bread [73]. As a consequence, core temperatures of the bread do not often exceed 90 °C during baking, thus favoring spore survival [48]. Spore germination and outgrowth are further enhanced when bread loaves are stacked closely together after baking, providing warmer and more humid conditions during the slower cooling of the loaves [22].

Temperature kinetics revealed that the bread core temperature increases to 100 °C in the first 15 min of baking. Due to the moisture evaporation–condensation mechanism, the bread crumb temperature becomes stable slightly below 100 °C, regardless of the oven temperature [19,75]. The rate by which bacterial spores are inactivated is time- and temperature-dependent, and heat resistance can be quantified by the decimal reduction time D at 100 °C (D_100_). Great variation, not only in species but also at the strain level, was observed regarding heat resistance of rope-spoilage-associated spore formers [19]. Leuschner et al. [59] assessed D_100_ values for spores of *B. subtilis*, *B. pumilus*, and *B. licheniformis* isolated from ropy partially baked bread dough at 14 min, 10 min, and 56 min (Table 1). Species assignment must be taken with caution, however, as API 50CH test kits were used for species identification [59]. Spore D_100_ values determined for *B. amyloliquefaciens* strains isolated from ropy bread varied from 23–45 min. Some strains belonging to the *B. cereus* group isolated from wheat flour had D_100_ values higher than 10 min (Table 1) [19].

Large intra-species variation in *Bacillus*-endospore heat resistance was attributed to the presence of the *spoVA^2mob^* operon in its genome [76,77]. Li, Schottroff, Simpson, and Gänzle [78] suggested that heat resistance and, consequently, *Bacillus* bread-spoilage potential could be tied to the presence of the *spoVA^2mob^* operon. The *spoVA^2mob^* operon was believed to be a suitable genetic marker for the identification of spoilage-associated spores because the authors found a positive correlation of the *spoVA^2mob^* operon copy number with the pressure resistance of *Bacillus* endospores [78]. Li et al. [78] further linked the presence of multiple copies of the *spoVA^2mob^* operon to increased heat stability. It might, therefore, be hypothesized that strains possessing multiple copies of the *spoVA^2mob^* operon are more prone to survive baking; however, Li et al. [74] showed that the *spoVA^2mob^* operon does not represent a prerequisite for *Bacillus* strains to survive baking. In fact, the presence of multiple copies of the *spoVA^2mob^* operon only exhibited a limited effect on survival during the baking procedure. Li et al. [74] rather associated the bread-spoiling activity of *Bacillus* spp. with the presence and expression of several extracellular amylases and proteases.

Thompson et al. [23] were not able to show a correlation between amylase activity and rope production. Further, all the rope-producing *Bacillus* strains isolated in the previously described study by Pepe et al. [25] exhibited amylase activity, but only 32.4% of these isolates were able to produce ropiness in bread slices after treatment of 96 °C for 10 min. When the strains were heated to 100 °C, no ropiness was observed [25].

The type and rate at which rope spoilage occurs is influenced by the type of bacterial strain and its endospore heat resistance, as well as its spoilage ability. A number of intrinsic and extrinsic factors related to the food and its storage, such as loaf type, pH, aw, microbiological species, and potential antagonisms between species (Figure 2B), also play a role, and the type and quantity of produced enzymes may vary in different strains of the same species or even within the same strain under different cultural conditions [19,23]. Therefore, high cell concentrations of spore-forming bacteria in bread do not automatically result in rope spoilage [19,79,80]. Valerio et al. [81] compared two *B. amyloliquefaciens* wild-type strains with *B. amyloliquefaciens* ATCC 8473 in order to assess their growth behavior and spoilage risk during bread shelf life. The results of this study highlight a higher rope spoilage potential in the isolated wild-type strains compared with the commercially available ATCC strain [81].

## 5. Prevention of Rope Formation in Bread

A combination of effective cleaning and sanitation practices with high-quality raw materials diminishes the incidence of rope spoilage, as contamination of dough should not exceed 1.0 CFU/g [12,82]. Since rope formers are very sensitive to low pH values, ropiness can be prevented by the addition of preservatives (Figure 2B) such as propionic acid, calcium propionate, acetic acid, and calcium hydrogen phosphate [12]. Pattison et al. [83] assessed the in vitro responses to acetic acid, lactic acid, calcium lactate, and a lactate-containing cocktail of *B. subtilis*, *B. pumilus*, and *B. licheniformis* isolates from ropy bread, under optimum growth conditions in broth media. The organic acids used in the study completely inhibited the growth of all tested *Bacillus* strains. When the pH was adjusted to the pH corresponding to baked brown bread containing the same preservative agents, efficacies decreased but were still as effective as calcium propionate, a common preservative in the baking industry [83]. Consistent with these findings, Pereira et al. [41] demonstrated that the addition of calcium propionate, together with low aw and pH, inhibited rope development in laboratory-baked bread inoculated with a *B. licheniformis* strain isolated from wholemeal flour and stored at 37 °C throughout a shelf life of seven days [41].

A frequently used natural preservative of bread is the addition of sourdough (Figure 2B), whose microbiota is dominated by lactic acid bacteria (LAB) [84]. Due to the production of lactic and acetic acids by LAB, the bread pH drops drastically, thus mostly inhibiting the growth of the *Bacillus* genera. In addition, some LAB secrete other antimicrobial compounds, such as bacteriocins, ethanol, hydrogen peroxide and fatty acids, that suppress *Bacillus* growth in the dough [85,86,87,88]. Hence, numerous studies assessing the inhibiting properties of LAB against *Bacillus* spp. and consequent rope formation were conducted [82,88,89,90,91,92,93]. The addition of 20% sourdough with pH > 4 to bread dough prevented the formation of visual rope by *B. subtilis* and *B. licheniformis* [94]. When the added sourdough had a pH of between 3.5 and 4, just 15% of sourdough was needed to prevent visible rope formation [93]. Interestingly, breads prepared with the same amounts of lactic acid as the dough containing 20% sourdough did not prevent *Bacillus* growth and subsequent rope development, suggesting that rope formation is prevented by the combination of reduced pH and low-molecular-mass compounds produced by LAB [94,95]. Mantzourani et al. [70] studied sourdough breads prepared with kefir grains, which showed good results against rope spoilage by *Bacillus* spp. It is believed that the organic acids play a synergetic role together with the antimicrobial compounds produced by LAB [92]. Another type of antagonistic bacteria used as a natural alternative to chemical food additives are propionic acid bacteria. As the name suggests, these bacteria have the innate ability to produce considerable quantities of propionic acid, thereby inhibiting *Bacillus* spp. based on pH reduction due to the synthesis of propionic acid [9,96].

Sudha et al. [97] reported the use of carambola-pomace powder in wheat bread to control the growth of rope-forming bacteria. The authors reported an equal minimum inhibitory concentration and minimum bactericidal concentration of 1.25 mg/mL for *B. spizizenii* ATCC 6633 and *B. cereus* ATCC 11,778 [97]. However, the addition of carambola fruit pomace powder is not suitable for the production of conventional white wheat bread, as the bread quality is significantly reduced. Breads baked with pomace powder have a decreased loaf volume, denser and more compact crumb of a brownish color, and a fruity to sour taste [97]. It is, therefore, not likely to be widely accepted by consumers.

## 6. Food Safety Concerns

As described in the sections above, *B. cereus* group members are commonly isolated from flours and are also implicated in ropy bread spoilage [25]. *B. cereus* is responsible for two foodborne gastrointestinal illnesses. While the emetic type is caused by the emetic toxin cereulide and leads to nausea and vomiting, the enteropathogenic type leads to diarrhea and abdominal pain and is caused by the toxins hemolysin BL (Hbl), nonhemolytic enterotoxin (Nhe), and cytotoxin K (CytK) [98]. In 2020, bacterial toxins accounted for 17.1% of all foodborne outbreaks in the European Union. Out of these, *Bacillus* toxins were the most frequently reported cause, with 71 cases. All cases were associated to the *B. cereus* group, except for one that was linked to *B. subtilis* [99]. Several authors published extensive reviews emphasizing food-safety concerns associated with the *B. cereus* group [98,100]. Thus, the *B. cereus* group should not be disregarded in the context of ropy bread spoilage, as agricultural products, such as cereals, are commonly contaminated by members of the *B. cereus* group [101].

In addition to the *B. cereus* group, there are other *Bacillus* species capable of toxin production [102]. Consequently, rope-forming bacteria may be hazardous, since bread is consumed by people of all ages, as well as people with various pre-existing conditions and hospitalized patients with various illnesses. For such people, bread containing high numbers of *Bacillus* spp., even those outside the *B. cereus* group, can be considered to be unsafe [103]. High counts of *B. subtilis* or *B. licheniformis* may cause diarrhea or vomiting and were implicated in foodborne outbreaks [41]. For example, *B. licheniformis*, *B. subitilis*, *B. mojavenis*, and *B. pumilus* produce toxins, such as lichenysin A, amylisin, and pumilacidin. Further, *B. amyloliquefaciens* is also known to produce amylosin [102]. In addition, the presence of toxigenic determinants, such as genes belonging to the Hbl and Nhe complex, were found in isolates belonging to the *B. subtilis* group, *B. licheniformis*, *N. circulans*, and *C. firmus* [102,104]. De Bellis et al. [102] examined the toxigenic potential and heat survival of spore-forming bacteria belonging to the genera *Bacillus*, and isolated from bread and its raw ingredients, mainly durum wheat semolina. Their results show that *B. amyloliquefaciens* strains almost completely survived heat treatment and retained cytotoxic activity. Although cytotoxicity was generally lower, it was detected in 30% of strains belonging to species other than the *B. cereus* group, including *B. amyloliquefaciens*, *B. mojavenis*, and *Peribacillus simplex* [102].

## 7. Considerations for the Future

The lack of systematic approaches allowing the characterization and quantification of rope formation makes assessment of rope development in bread extremely difficult. Further, models in which bread slices are directly inoculated with bacteria previously grown in BEB media are tedious. Aside from spores, viable cells and growth media are also spread onto bread slices, but to better model reality, slices should be merely inoculated with spores. Furthermore, a combination of methods will likely reveal meaningful information about the actual behavior of *Bacillus* spp. in bread. For instance, Pereira et al. [41] used direct inoculation of bread slices for pre-screening potential rope-forming strains and baking trials of one selected isolate to investigate its rope-forming potential in different dough formulations. Li et al. [74] also applied a combination of baking and direct inoculation of bread slices with *Bacillus* spores. The authors relied on baking trials to determine the survival of *Bacillus* spp. with different copy numbers of the *spoVA^2mob^* operons. Bread slices were afterwards directly inoculated with spores to determine the spoilage phenotype of different *Bacillus* strains [74]. Both studies acknowledge the importance of this combination, but the characterization and quantification of rope formation was still conducted by somewhat subjective parameters, given the development of patchy discoloration, bread-crumb deterioration, and the characteristic smell produced by rope-forming strains. Thus, more objective parameters are needed to improve the characterization of rope-forming bacilli. Because the attributes of bread-crumb discoloration and deterioration are considered strain dependent, an objective quantification of parameters, such as texture and color, would be of great importance.

The lack of standard protocols and interpretation methods for the analysis of rope spoilage ultimately leads to the absence of clear boundaries for a proper definition of ropiness in bread, and the identification of rope-forming strains and non-rope forming strains, respectively. For instance, hard evidence of enhanced extracellular amylase and protease expression in rope-forming strains compared to non-rope forming strains is still to be found. Furthermore, genetic data on rope formers are scarce and, therefore, the genetic basis of rope formation is still poorly understood. The development of standard protocols and the generation of whole-genome sequence data of rope formers and strains not able to cause rope formation could enable the identification of biomarkers useful for the prediction of RIP. Detection procedures for quick identification of rope-forming bacilli could subsequently be developed to enhance quality control within the baking industry.

As software development and computational power increased rapidly over the last years, many mathematical models for quantitative microbial risk assessment were rendered [105,106]. These models assess, for example, the growth kinetics of foodborne pathogens, such as *Listeria monocytogenes*, in ready-to-eat foods, *Salmonella enterica* in eggs, and *B. cereus* in fried rice [105,107]. Models to optimize baking processes and assess the relationship of raw ingredients and physicochemical properties of baked bread, such as its volume or crumb texture, were also created [105,108,109,110]. The design of predictive models to estimate rope development in bread, as created for the assessment of the spoilage potential of *Clostridium tyrobutyricum* during cheese ripening, would be of great value [111]. As a prerequisite for predictive models, a deeper understanding of rope spoilage and comprehensive data are required.

## 8. Conclusions

Even though rope spoilage in bread is a long-studied phenomenon, its exact causative mechanisms and genetic background are not completely understood. While various studies assessed the prevalence of different rope-forming *Bacillus* spp. in bread and its raw materials, many aspects involved in this complex phenomenon are still poorly understood, as standardized, objective classification protocols are missing, and many studies used tools for species identification that are not able to reliably differentiate closely related *Bacillus* species. The rise in consumer demand for preservative-free, whole grain foods and the rising ambient temperatures caused by global warming may favor the occurrence of rope spoilage in bread. Further research elucidating the dynamics behind this spoilage phenomenon is urgently needed to support high-quality bread production and prevent food waste.

## Figures and Tables

**Table 1:** Bacterial species that were suggested to cause rope spoilage in bread. The table summarizes data on growth characteristics, metabolism, and spore survival.

	Growth						Metabolism						Survival	
Taxonomy	Optimum Growth Temperature [°C]	Minimum Growth Temperature [°C]	Maximum Growth Temperature [°C]	Growth at pH	NaCl Tolerance	Anaerobic Growth	Urease	Nitrate Reduction	Hydrolysis of Starch	Citrate	Propionate	Egg Yolk Reaction	Spore D_100_ Value [min]	References
***B. amyloliquefaciens***	30–40	15	50	5.7	5–10%	−	+	d	+	+	n.a.	+	23–44	[19,26,50,51,52,57,58,59]
***B. cereus* group**	37	5	50	4.9–9.3	n.a.	+	d	+	+	d	n.a	+	<10	[24,26,40,41,41,42,43,44,45,46,47,48,49,50,51,52,60,61]
***B. licheniformis***	37	15	50–55	5.7	7%	+	+	+	+	+	+	−	56	[19,24,26,41,50,51,52,59,60,62,63,64]
***B. pumilus***	30	15	50–55	5.7	7%	+	+	+	+	+	+	−	56	[19,24,26,41,50,51,52,59,60,65]
***B. sonorensis***	30	15	55	n.a.	<5%	+	+	+	+	+	+	−	n.a.	[41,60]
***B. subtilis* ^a^**	28–30	5-20	45–55	5.5–8.5	7–10%	Facultative	−	+	+	+	−	−	14	[7,15,24,25,26,41,50,51,52,53,54,60,63,66]
***C. firmus* ^b^**	30–37	n.a.	n.a.	n.a.	n.a.	n.a.	−	+	+	−	n.a.	n.a.	n.a.	[50,51,52]
***N. circulans* ^c^**	30–37	n.a.	n.a.	n.a.	n.a.	n.a.	−	n.a.	+	−	n.a.	n.a.	n.a.	[50,51,52]
***P. polymyxa* ^d^**	30	n.a.	n.a.	n.a.	n.a.	n.a.	−	n.a.	n.a.	n.a.	n.a.	n.a.	n.a.	[26,41,60]
***Pr. megaterium* ^e^**	30	3-15	35–45	n.a.	7%	−	+	d	+	+	n.a.	−	n.a.	[26,52,65]

The symbol + denotes positive, − negative, and d diverse results in the respective tests/compound utilization. When no data were available, this is indicated by n.a. ^a^ *B. inaquosorum*, *B. spizizenii*, and *B. stercoris* formerly subsumed under the species *B. subtilis,* were recently designated as separate species, according to Dunlap et al. (2020) [53]. *B. subtilis* isolates that were involved in rope spoilage, could potentially belong to these new species. However, there is no direct evidence of their involvement in rope spoilage. Metabolic characteristics may differ from *B. subtilis*. ^b^ formerly designated as *B. firmus*. ^c^ formerly designated as *B. circulans*. ^d^ formerly designated as *B. polymyxa*. ^e^ formerly designated as *B. megaterium*.
